# Supplementary material for: The Efficacy of CB-103, a First-in-Class Transcriptional Notch Inhibitor, in Preclinical Models of Breast Cancer
Source: Cancers (Basel). 2023 Aug 3;15(15):3957. doi: 10.3390/cancers15153957 (PMC10416998; doi:10.3390/cancers15153957)
Supplement: Supplementary file 1 [file cancers-15-03957-s001.zip › cancers-2527665-supplementary.pdf]

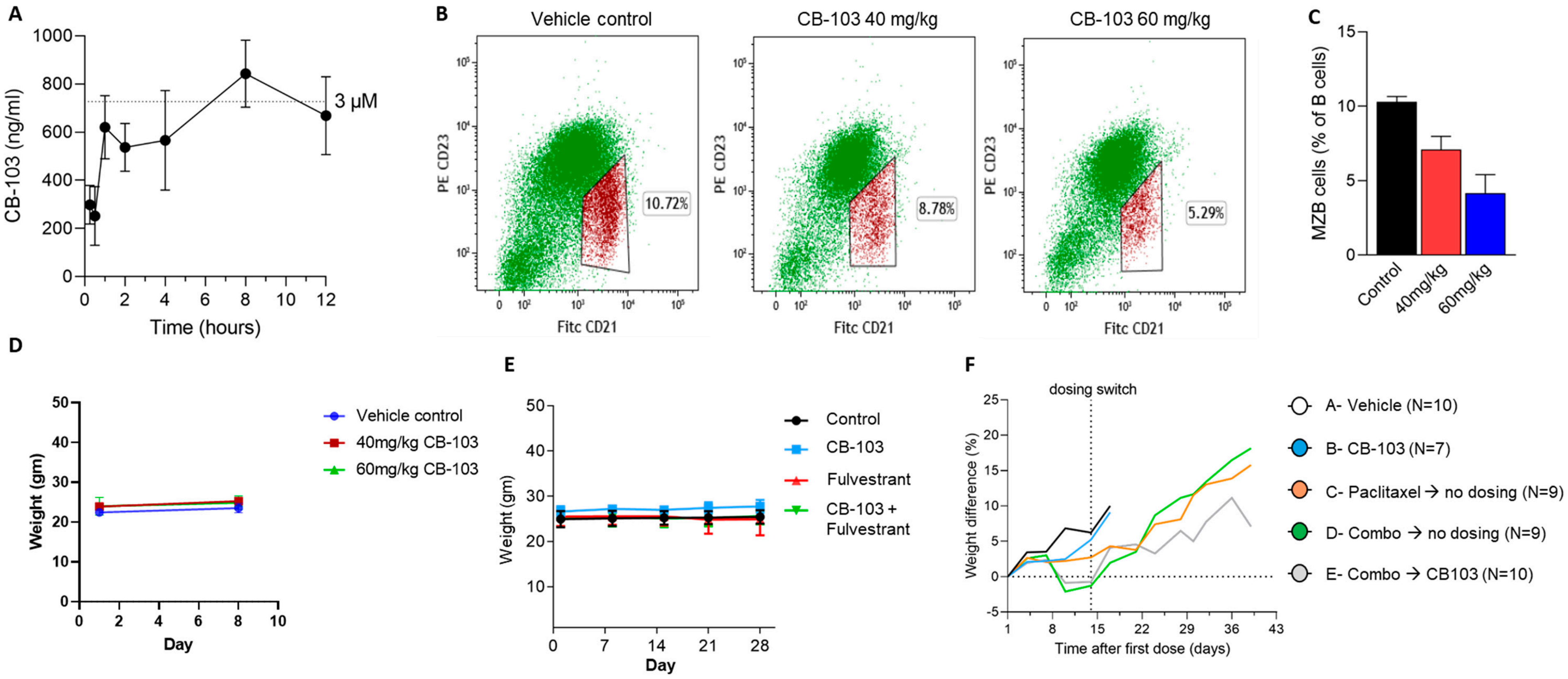

Supplementary Figure S1. Pharmacokinetics, pharmacodynamics and weight of mice during in vivo studies. (**A**) Plasma concentration of CB-103 at 0.25, 0.5, 1, 2, 4, 8, 12 hours after single dose at 60 mg/kg. Each dot represents mean ± SEM of n=3 mice. (**B**) Dose dependent downregulation of Marginal Zone B (MZB) cells in mice upon 7 days of CB-103 daily dosing; flow cytometry data showing percentage of MZB cells of representative mouse from vehicle control, 40 mg/kg and 60mg/kg of CB-103 treatment; (**C**) Histogram plot of the mean ± SEM frequency of MZB cells for each dosing group (n=4). (**D**) Weight plot of mice in the target engagement cohort in the MZB cell experiment. (**E**) Weight chart of mice in the Fulvestrant efficacy cohort indicating tolerability of doses. (**F**) Weight chart of mice in the paclitaxel efficacy cohort indicating tolerability of doses.

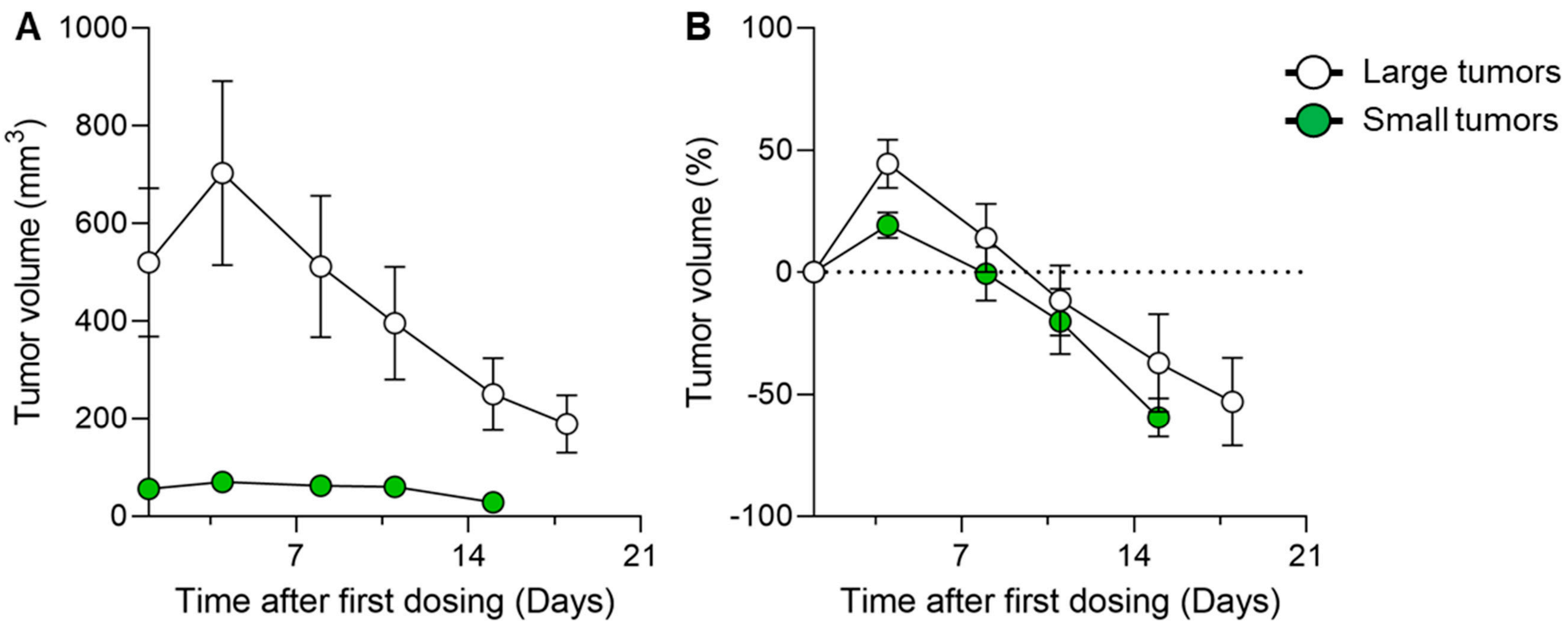

Supplementary Figure S2. Efficacy of CB103 + paclitaxel therapy on small and large TNBC tumors. NSG immune compromised female mice engrafted subcutaneously with TNBC cell line HCC1187 were treated with combination therapy (CB-103 60 mg/kg QDx5 + Paclitaxel 10 mg/kg Q7D) once average tumor volume reached 50 mm^3 (green dots) or 500 mm^3 (white dots). Tumor growth was evaluated by measuring tumor volume (A) or the percentage difference of volume from beginning of dosing (B).

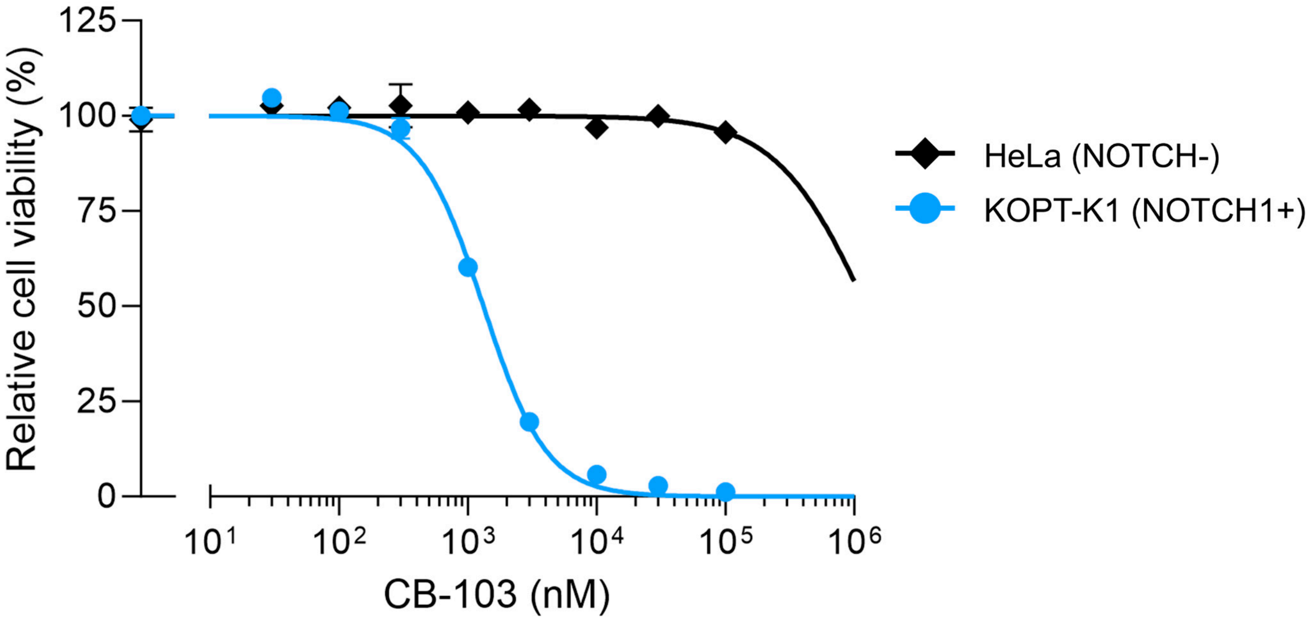

Supplementary Figure S3. CB-103 specifically affects the viability of NOTCH+ cell lines. CB-103 was titrated (30 nm - 100 µM) on NOTCH1-positive KOPT-K1 cells or on NOTCH-negative HeLa cells (negative control) for four days and the viability of each condition was measured by PrestoBlue™ assay. Each dot represents mean ± SD of 3 technical replicates. The line represents the dose-response fit generated by GraphPad Prism 10.0 using log(inhibitor) vs response and variable slope (four parameters) setting.
